# Abacavir enhances the efficacy of doxorubicin via inhibition of histone demethylase KDM5B in breast cancer

**DOI:** 10.1038/s41598-025-13845-z

**Published:** 2025-08-05

**Authors:** Anmi Jose, Pallavi Kulkarni, Naveena AN Kumar, Nawaz Usman, Gabriel Sunil Rodrigues, Gautham G. Shenoy, Rama Rao Damerla, Murali Munisamy, Bharti Bisht, Sooryanarayana Varambally, Manash K. Paul, Neha Arya, Praveen PN Rao, Mahadev Rao

**Affiliations:** 1 Department of Pharmacy Practice, Manipal College of Pharmaceutical Sciences, Manipal Academy of Higher Education, Manipal, Karnataka 576104 India; 2 Department of Biochemistry, All India Institute of Medical Sciences Bhopal, Bhopal, Madhya Pradesh 462020 India; 3 Department of Surgical Oncology, Manipal Comprehensive Cancer Care Centre, Kasturba Medical College, Manipal Academy of Higher Education, Manipal, Karnataka 576104 India; 4 Department of General Surgery, Kasturba Medical College, Manipal Academy of Higher Education, Manipal, Karnataka 576104 India; 5 Department of Pharmaceutical Chemistry, Manipal College of Pharmaceutical Sciences, Manipal Academy of Higher Education, Manipal, Karnataka 576104 India; 6 Department of Medical Genetics, Kasturba Medical College, Manipal Academy of Higher Education, Manipal, Karnataka 576104 India; 7 Department of Translational Medicine, All India Institute of Medical Sciences Bhopal, Bhopal, Madhya Pradesh 462020 India; 8 Department of Microbiology, Kasturba Medical College, Manipal Academy of Higher Education, Manipal, Karnataka 576104 India; 9 Department of Pathology, University of Alabama at Birmingham, Birmingham, AL USA; 10 Department of Radiation Biology and Toxicology, Manipal School of Life Sciences, Manipal Academy of Higher Education, Manipal, Karnataka 576104 India; 11 School of Pharmacy, Health Sciences Campus, University of Waterloo, 200 University Avenue West, Waterloo, ON N2L 3G1 Canada

**Keywords:** Breast cancer, Carbovir triphosphate, Drug repurposing, Epigenetic targeting, Precision medicine, Breast cancer, Cancer therapy, Oncogenes, Tumour biomarkers, Preclinical research, Translational research, Cancer, Computational biology and bioinformatics, Biomarkers, Pathogenesis

## Abstract

KDM5B, a lysine-specific histone demethylase, is widely upregulated in breast cancer. The current study investigated the role of KDM5B in breast cancer and explored the repurposing potential of the antiviral drug abacavir (ABC). The cytotoxic effects and the effect of ABC sensitization on doxorubicin (DOX) efficacy were evaluated using 2-D and 3-D cell culture models. KDM5B expression was elevated in breast cancer tissues compared to normal breast tissues. In vitro studies demonstrated that ABC treatment reduced KDM5B expression in breast cancer cells and increased their sensitivity towards DOX. ABC induced late apoptosis and S-phase arrest, while the ABC + DOX combination led to S/G2 phase arrest, late apoptosis, and cell death. Data generated from patient-derived breast tumoroids corroborated the 2-D cell culture-based findings. Additionally, molecular docking studies indicated that the active drug metabolite carbovir triphosphate (CBV-TP) could interact with the DNA polymerase β-DNA complex, suggesting its potential mechanism to be incorporated into the DNA synthesis cycle, leading to cell cycle arrest in tumor cells. Our findings highlight the repurposing potential of ABC to target KDM5B in breast cancer. This approach enhanced the efficacy of DOX, which could allow further dose reduction and reduced side effects, offering a promising therapeutic strategy.

**Supplementary Information:**

The online version contains supplementary material available at 10.1038/s41598-025-13845-z.

## Introduction

Breast cancer remains the most prevalent cancer among women worldwide, with an estimated 2.2 million new cases and 0.6 million deaths reported in 2022^1^. Tumor heterogeneity in breast cancer results in significant variability in pathology, molecular alterations, and tumor microenvironment^2^. While the development in early diagnosis and treatment modalities has improved overall survival rates of breast cancer, the effective management of breast cancer still faces challenges^3^. Since breast cancer is influenced by a combination of genetic and epigenetic factors, a detailed understanding of the underlying molecular mechanisms is necessary to improve therapeutic options. Although epigenetic modifications do not cause alterations in DNA sequences, they can significantly influence cancer development at different stages. Hence, targeting epigenetic markers has a substantial role in cancer detection, prevention, and development of targeted therapies^4^. For example, drugs targeting epigenetic modifying enzymes, such as histone deacetylases (HDACs) and DNA methyltransferases, have received FDA approval^5^. Furthermore, combining epigenetic targeting drugs (e.g., decitabine, SAHA, etc.) with conventional chemotherapies has also received wide attention^6–8^.

KDM5B (Lysine demethylase 5B), or JARID1B, encodes lysine-specific histone demethylase from the Jumanji (JmjC)/ARID domain-containing family of histone demethylases^9^. This protein plays a pivotal role in the transcriptional repression of genes by demethylating mono-, di-, and trimethylated lysine 4 of histone 3 (H3K4)^10^. KDM5B is significantly upregulated in various cancers, particularly breast cancer^11,12^, and its oncogenic properties make it a promising target for personalized drug therapy^13^. However, the prognostic significance and drug-targeting potential of KDM5B have not been fully explored. In this context, repurposing existing drugs could be a valuable strategy, owing to the established safety profile, cost-effectiveness, shorter development times, and affordability^14^.

Several nucleoside derivatives with monocyclic or bicyclic ring systems have been reported to possess anticancer properties^15^. With this rationale, we screened a panel of nucleoside-based compounds as inhibitors of KDM5B^16^. Abacavir (ABC), a nucleoside reverse transcriptase inhibitor (NRTI), is commonly used in the treatment of human immunodeficiency virus type 1 (HIV-1) infection. ABC is generally well tolerated with minimal adverse effects and favourable bioavailability^17^. Upon cellular uptake, ABC undergoes bioconversion to form the active metabolite carbovir triphosphate (CBV-TP)^18^. In a previous study, molecular docking analysis identified the key interactions of ABC towards the JmjC domain of KDM5B^16^. Furthermore, former studies have demonstrated the potential of ABC as an anticancer agent in various malignancies^19,20^. In this regard, we conducted a detailed analysis to evaluate the repurposing potential of ABC by targeting the KDM5B oncogene in breast cancer. KDM5B gene expression was determined in breast cancer patient samples, and the cytotoxic activity and the effect of ABC sensitization on doxorubicin (DOX) were investigated in 2-D and 3-D cultures. The results from our study demonstrated that ABC could potentiate the cytotoxicity of DOX in breast cancer cells. This approach enhanced the efficacy of DOX, which could allow for further dose reduction, leading to lower side effects, thus offering a promising strategy for breast cancer therapy.

## Results

### KDM5B is over-expressed in breast cancer: evidence from public databases

**Fig. 1:**
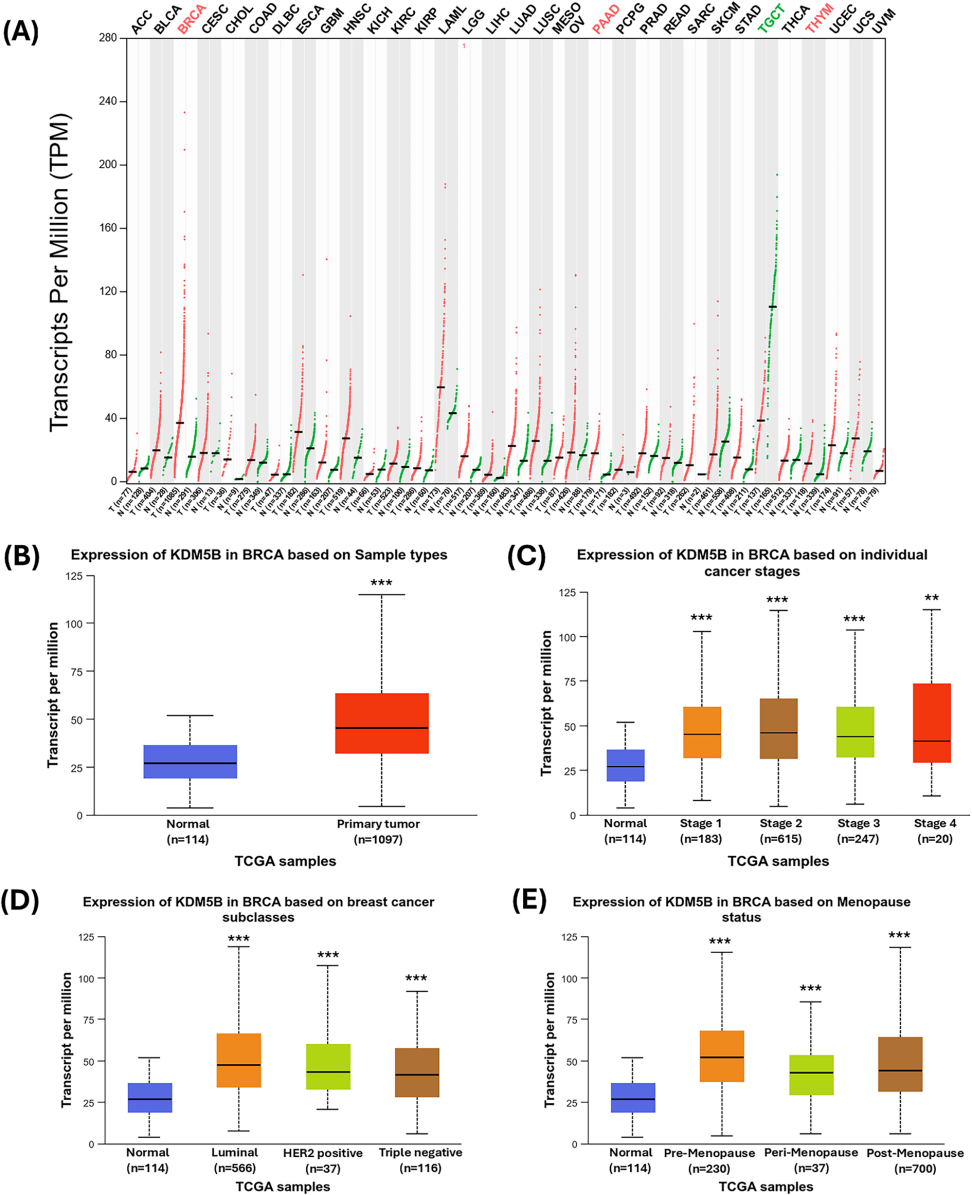
KDM5B expression levels in different human tumors and breast cancer; (**A**) GEPIA database-based expression of KDM5B in different tumors compared to normal tissue. The horizontal axis cancer name in red indicates high expression, green indicates low expression, and black indicates no statistical significance; (**B**–**E**) Analysis of expression profiles of KDM5B in breast cancer across different sample types (**B**), stages (**C**), subclasses (**D**), and menopausal status (**E**) using UALCAN database; *P < 0.05; **P < 0.01; ***P < 0.001.

Comprehensive gene expression profiling of KDM5B across tumor and normal tissues was evaluated using the GEPIA database^21,22^. The KDM5B gene expression was elevated in most cancers compared to normal tissues, with significant overexpression observed in breast invasive carcinoma, pancreatic adenocarcinoma, and thymoma (Fig. 1A). Further analysis using UALCAN database^23–25^ confirmed that KDM5B gene expression was significantly higher in breast tumors than in normal breast tissues (Fig. 1B). In addition, RNA-Seq data from the TNMplot database^26,27^ demonstrated increased KDM5B expression in both primary breast tumors and metastatic breast cancer samples compared to normal breast tissue (see Supplementary Fig. S1 online). While KDM5B expression is elevated in all tumor stages compared to normal tissue, early-stage tumors exhibit a slightly higher median expression than advanced-stage tumors (Fig. 1C). Across all breast cancer subtypes, KDM5B expression was marginally higher in the luminal subtype, followed by HER2 positive and triple negative (Fig. 1D). Menopausal status also correlated with KDM5B expression. Patients in the premenopausal stage showed significantly higher KDM5B levels compared to those in the perimenopausal, and post-menopausal stages (Fig. 1E). Additional histopathological and clinical parameters, including patient’s age, race, nodal metastasis, histologic subtypes, and TP53 mutation status, were significantly associated with KDM5B overexpression (see Supplementary Fig. S1 online). Overall survival (OS) in breast cancer patients with KDM5B mRNA expression was analyzed using the GEPIA database (see Supplementary Fig. S2 online). KDM5B expression was not significantly associated with OS in breast cancer patients.

### KDM5B is over-expressed in clinical breast tumor samples

**Fig. 2:**
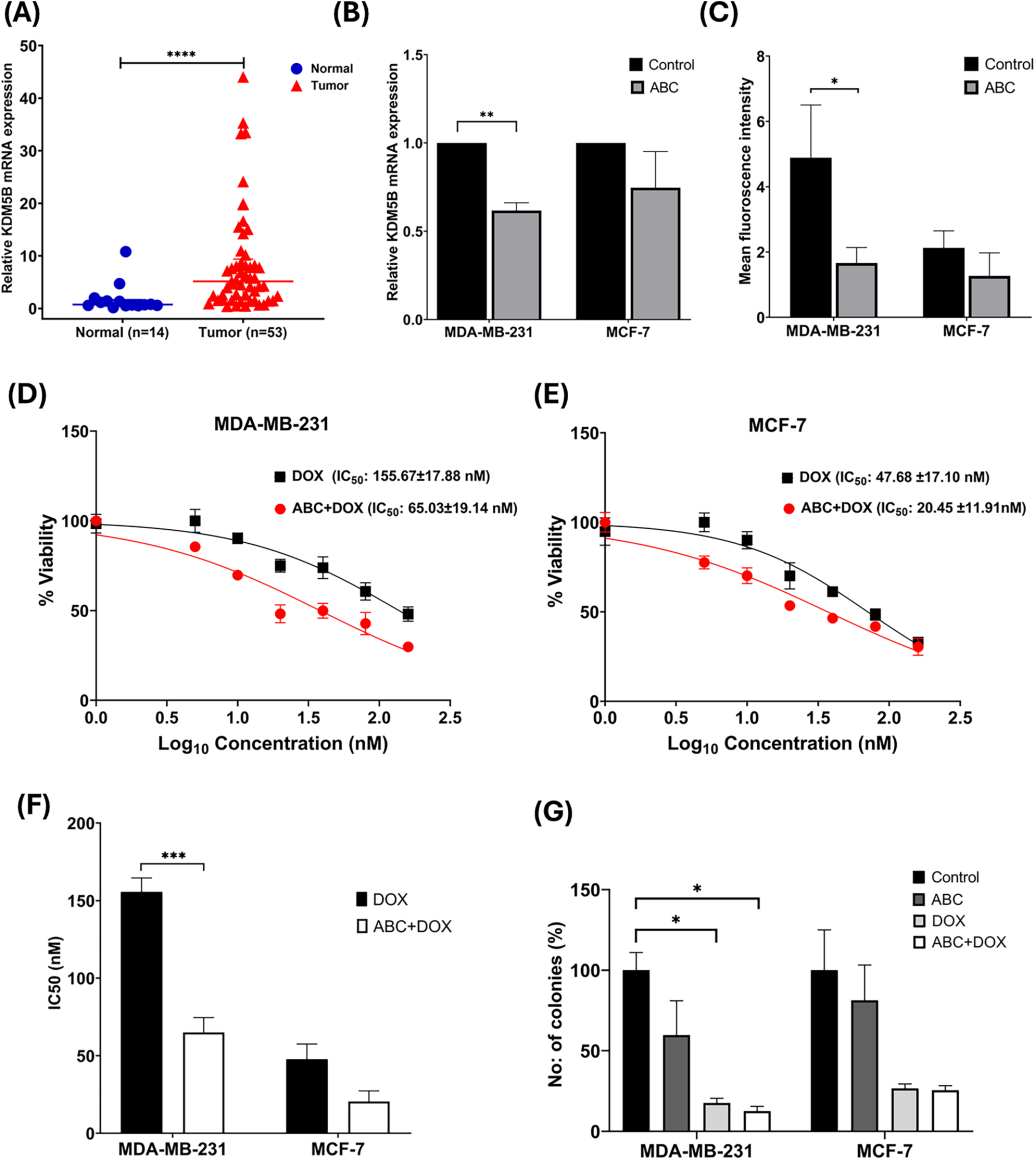
(**A**) KDM5B mRNA expression levels in normal (*n* = 14) and breast tumor tissues (*n* = 53); (**B**) KDM5B mRNA expression following ABC treatment (IC_25_) for 24 h in MDA-MB-231 and MCF-7 cells; (**C**) Quantification of KDM5B mean fluorescence intensity in control and treated MDA-MB-231 and MCF-7 cells; (**D**,**E**) Representative dose response curves from MTT assay showing enhanced DOX cytotoxicity upon ABC sensitization in (**D**) MDA-MB-231 and (**E**) MCF-7 cells; IC_50_ values represent the mean ± SD of three independent experiments (**F**) Bar graph showing IC_50_ values of DOX with or without ABC sensitisation in both cell lines. Data is expressed as mean ± SD of three independent experiments; (**G**) Soft agar colony formation assay comparing untreated control and three treatment groups in MDA-MB-231 and MCF-7 cells. Bar graph data are expressed as mean ± SD from three independent experiments. *P < 0.05; **P < 0.01; ***P < 0.001; ****P < 0.0001.

The results obtained in databases were validated in clinical tumor samples. Towards this, qPCR was performed on 53 breast cancer and 14 normal breast tissue samples to evaluate KDM5B expression. In line with global data, KDM5B expression was significantly elevated in breast tumors compared to normal breast tissues (*P* < 0.0001) (Fig. 2A). However, no significant differences in KDM5B expression were observed across various clinicopathological factors. Detailed patient characteristics and clinicopathological data in relation to KDM5B mRNA expression are provided in (Table 1).

**Table 1:** Statistical analysis of KDM5B expression levels in clinical breast cancer tissues in relation to clinicopathological characteristics. *ER* estrogen receptor, *DCIS* ductal carcinoma in situ, *HER2* human epidermal growth factor receptor 2, *NST* no special type, *IQR* interquartile range, *PR* progesterone receptor, *TNBC* triple-negative breast cancer.

Clinicopathological features	Sample size (*n*)	KDM5B mRNA expression		
		Median	IQR (Q1, Q3)	*P* value
Sample type				
Tumor	53	5.138	1.610, 9.381	**< 0.0001**
Normal	14	0.740	0.557,1.556	
Age (years)				
≤ 50	27	5.809	2.588, 8.594	0.414
> 50	26	4.224	1.521, 14.850	
Tumor histology				
Invasive breast carcinoma NST	46	4.599	1.546, 10.36	0.847
Other (DCIS, Mixed, Metaplastic carcinoma, etc.)	7	5.809	2.405, 7.803	
Menopausal status				
Pre-menopausal	19	5.767	2.588, 8.594	0.733
Post-menopausal	34	4.402	1.523, 11.190	
Pathological grade				
Grade 1	7	2.702	1.417, 8.185	0.639
Grade 2	19	4.516	1.551, 15.040	
Grade 3	27	5.767	1.668, 10.170	
TNM stage				
I	5	2.405	1.365, 5.982	0.576
II	42	5.586	1.625, 11.780	
III	4	4.887	2.154, 7.596	
IV	2	10.65	1.529, 19.770	
Nodal involvement				
0	32	5.788	2.617, 10.760	0.150
I	13	2.405	1.116, 11.740	
II	3	1.417	0.693, 5.138	
III	5	8.192	3.927, 12.040	
ER				
ER+	36	5.854	2.351, 10.360	0.302
ER−	17	4.287	1.208, 8.662	
PR				
PR+	26	4.960	2.138, 8.287	0.978
PR−	27	5.138	1.529, 10.950	
HER2				
HER2+	12	5.453	2.292, 16.900	0.614
HER2−	40	4.846	1.564, 9.775	
Unknown	1	4.161	-	
Molecular subtypes				
Luminal A	10	5.784	2.387, 10.210	0.944
Luminal B	25	5.404	1.610, 9.572	
TNBC	12	5.048	1.063, 13.230	
HER2 enriched	5	4.681	1.525, 12.850	
Unknown	1	4.161	-	

### Repurposing of the antiviral drug ABC targeting KDM5B, sensitized breast cancer cells to DOX

We previously reported the potential of repurposing antiviral drugs targeting the JmjC domain of KDM5B through computational analysis, including molecular docking studies of ABC^16^. Furthermore, former literatures demonstrated the potential of ABC as an anticancer agent in various cancers^19,20^. To assess its therapeutic efficacy in breast cancer cells, we examined the cytotoxic effects of ABC at concentrations ranging from 0 to 500 µM in MDA-MB-231 and MCF-7 cells. Interestingly, ABC exhibited significant cytotoxicity at clinically relevant concentrations in both cell lines (MDA-MB-231 IC_50_: 289.15 ± 32.25 µM; MCF-7 IC_50_: 281.26 ± 51.59 µM) (see Supplementary Fig. S3 online).

In addition, to investigate whether ABC treatment modulates KDM5B mRNA expression, we analyzed transcript levels in ABC-treated cells. Cells were exposed to a low dose of ABC (IC_25_) for 24 h, followed by a 3-day media change. Compared to untreated control cells, KDM5B mRNA levels were reduced in both cell lines, with a significant decrease observed in ABC-treated MDA-MB-231 cells (*P* < 0.01) (Fig. 2B). Furthermore, to determine whether these changes were reflected at the protein level, KDM5B expression was assessed by immunofluorescence under similar treatment conditions. Consistent with the mRNA data, a reduction in KDM5B protein levels was observed in both MDA-MB-231 and MCF-7 cells compared to untreated controls, with a decrease reaching statistical significance in MDA-MB-231 cells (*P* < 0.05) (Fig. 2C and Supplementary Fig. S4 online).

Since ABC treatment influenced KDM5B expression at both transcript and protein levels and considering that KDM5B is known to promote cancer cell proliferation^12^, we further examined whether ABC could enhance the sensitivity of breast cancer cells to DOX treatment. Pre-treatment with a low dose of ABC (IC_25_ dose), followed by DOX exposure (0-160 nM), significantly enhanced cytotoxicity, reducing cell proliferation more efficiently than DOX alone (Fig. 2D and E). In the absence of ABC, MDA-MB-231 cells demonstrated enhanced resistance to DOX (IC_50_ = 155.67 ± 17.88 nM), compared to MCF-7 cells (IC_50_ = 47.68 ± 17.10 nM). However, in ABC-sensitised cells, the IC_50_ of DOX was significantly reduced to 65.03 ± 19.14 nM in MDA-MB-231 cells, representing a 2.4-fold decrease (*P* < 0.001), and to 20.45 ± 11.91 nM in MCF-7 cells, indicating a 2.3-fold reduction (*P* > 0.05), demonstrating enhanced cytotoxicity compared to DOX alone (Fig. 2F). Subsequently, soft agar colony forming assay was performed with three pretreated groups (ABC, DOX, ABC + DOX) along with an untreated control (Fig. 2G and Supplementary Fig. S5 online). In MDA-MB-231 cells, both DOX and ABC + DOX treatment significantly reduced colony formation compared to the untreated control. However, no statistically significant difference was observed between DOX and ABC + DOX groups. This may be due to the relatively low dose and limited duration of ABC sensitization, which may not have been sufficient to fully impair the clonogenic property of the cells.

### Pre-treatment of ABC followed by DOX enhanced apoptosis and cell cycle arrest in breast cancer cells

**Fig. 3:**
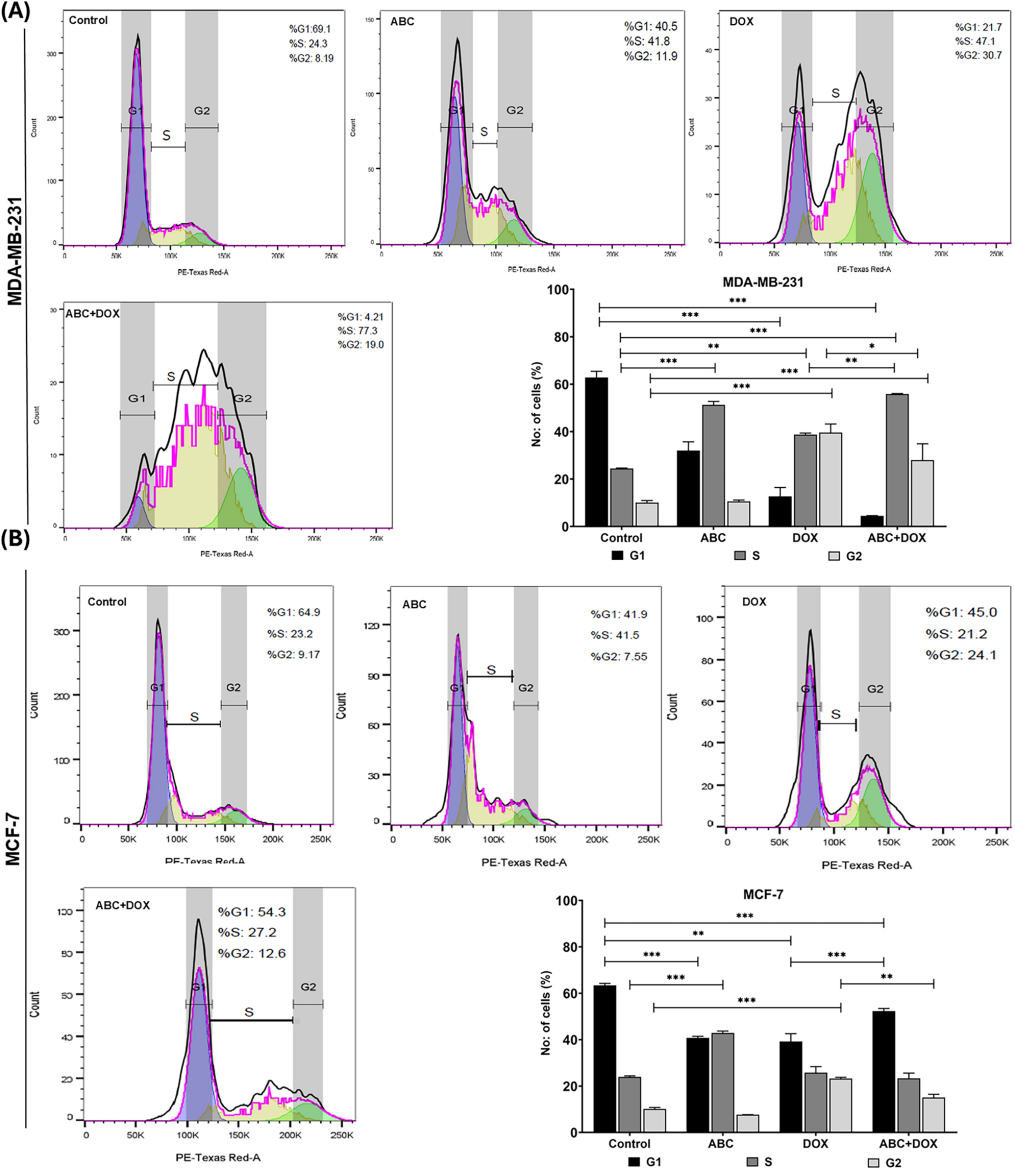
Representative images of cell cycle analysis by flow cytometry showing ABC treatment induced S phase arrest, whereas ABC + DOX had more cells in the S/G2 phase in (**A**) MDA-MB-231 and (**B**) MCF-7 cells. Bar graph data are expressed as the mean ± SD of three independent experiments. *P < 0.05; **P < 0.01; ***P < 0.001.

We investigated the effects of ABC on cell cycle progression and apoptosis in MDA-MB-231 and MCF-7 cells. Treatment with ABC alone for 72 h (IC_25_, added every 24 h) induced S-phase arrest in both cell lines (Fig. 3). Combined treatment with ABC (IC_25_ for 24 h) and DOX (IC_25_ for 72 h) resulted in an arrest at the S/G2 phase. A more evident cell cycle arrest with ABC sensitization was observed in MDA-MB-231 cells compared to MCF-7 cells. DNA replication occurs during the S phase, while the G2 phase prepares the cell for mitotic division. Arresting cells in the S/G2 phase indicates disruption of cell cycle progression, effectively inhibiting cell proliferation. Our findings suggest that, as ABC can induce DNA damage, it can lead to transient S/G2-phase arrest and apoptosis. This dual strategy of inducing S/G2 phase arrest and DNA damage represents an effective approach for inhibiting breast cancer cell proliferation and inducing cell death.

**Fig. 4:**
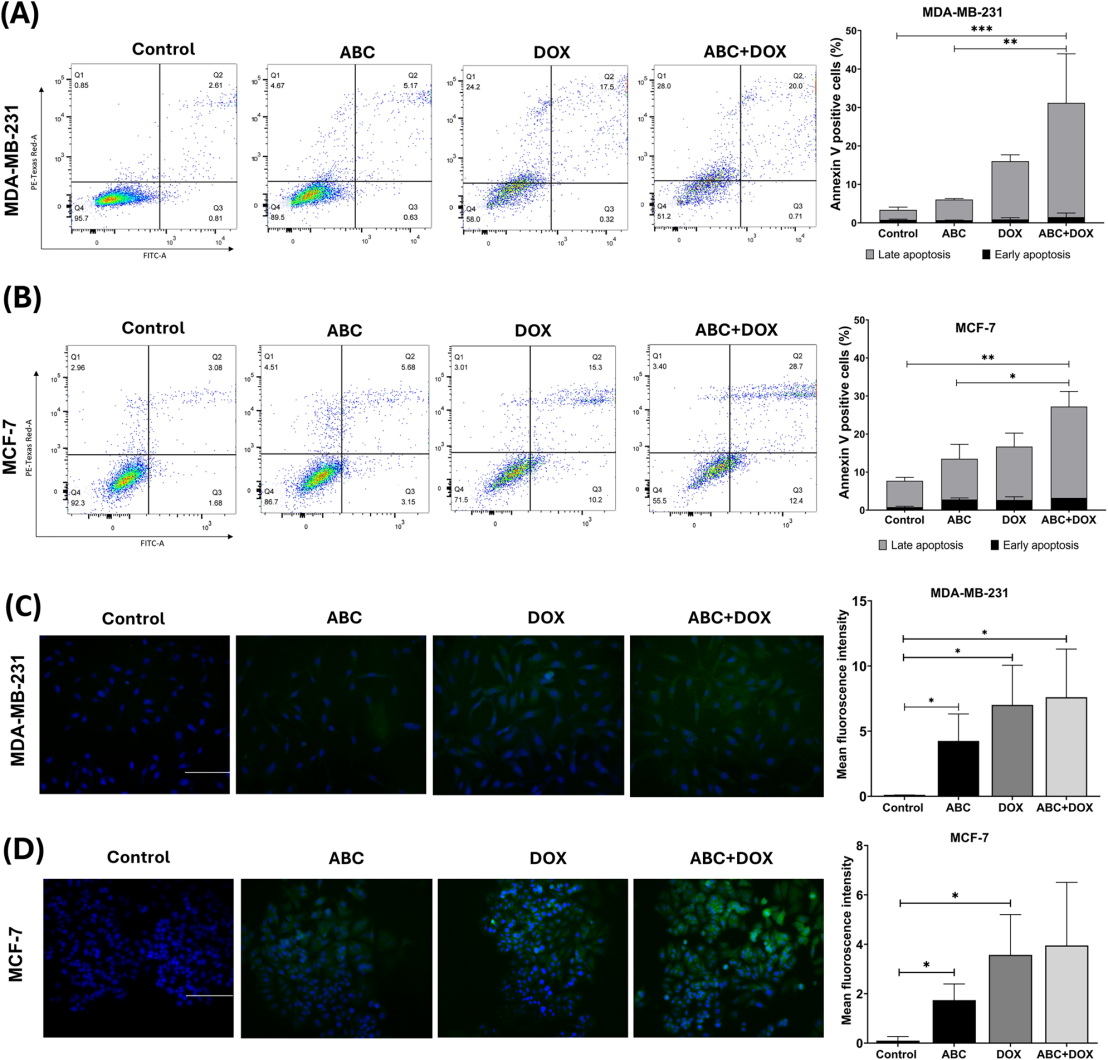
(**A**,**B**) Representative images of Annexin V/PI apoptosis study by flow cytometry showing ABC + DOX treatment-induced late apoptosis in (**A**) MDA-MB-231; (**B**) MCF-7 cells. Bar graph data are expressed as the mean ± SD of three independent experiments; (**C**,**D**) Representative images of the immunofluorescence staining profile of cleaved caspase-3 in control, ABC, DOX, and ABC + DOX treatment in (**C**) MDA-MB-231; (**D**) MCF-7. The bar graphs show the mean fluorescence intensity in control and treated cells. Expression was normalised to DAPI, and the data is represented as mean ± standard deviation; Scale bar: 500 μm. *P < 0.05; **P < 0.01; ***P < 0.001.

In addition, apoptosis analysis showed that treatment with ABC alone slightly increased the cell population undergoing late apoptosis in both cell lines, with a slight increase in early-phase apoptosis observed in MCF-7 cells. Combined treatment with ABC (IC_25_ for 24 h) and DOX (IC_25_ for 72 h) significantly increased the percentage of cells in late-phase apoptosis in both cell lines (Fig. 4A and B). To further validate these findings, we assessed cleaved caspase-3 expression levels in both cell lines (Fig. 4C and D). The results demonstrated a significant upregulation of cleaved caspase-3 in ABC-treated cells, corroborating the apoptosis-inducing effect of ABC observed in the Annexin V/PI assay. Consistent with the cytotoxicity data, a more pronounced apoptotic response was observed in MDA-MB-231 cells compared to MCF-7 cells.

### Generation of patient-derived breast cancer organoids

**Fig. 5:**
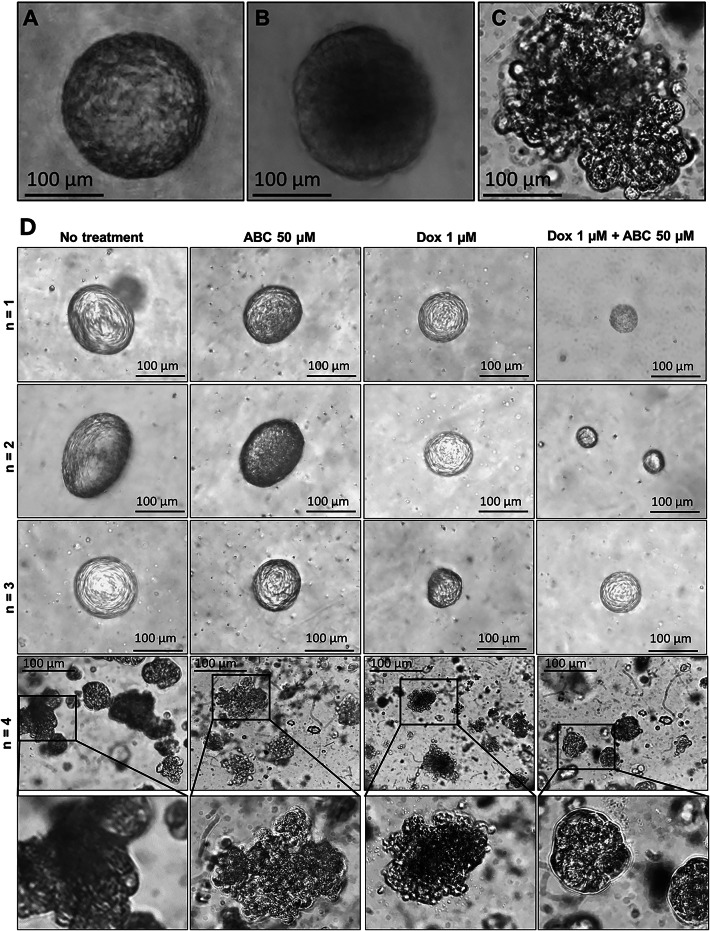
Representative bright-field images of patient-derived breast cancer organoid phenotypes. Two types of breast cancer organoids were observed in different patients: (**A**,**B**) Solid-dense and (**C**) Discohesive. (**D**) Sensitivity of patient-derived breast cancer organoids to ABC, DOX, and their combination. Representative bright field images of breast cancer organoids treated with ABC (50 µM) and DOX (1 µM). Scale = 100 μm. Patients 1, 2, 3, and 4 are represented as *n* = 1, *n* = 2, *n* = 3, and *n* = 4. The lower panel shows zoomed-up images of the discohesive breast cancer organoids.

Four patient breast cancer samples were obtained under informed consent and used to generate patient-derived breast cancer organoids, as described in the methods section. We observed two types of breast cancer organoid phenotypes, including solid dense (Fig. 5A,B) and discohesive (Fig. 5C)^28^. The solid-dense phenotype was associated with three patients, while the discohesive phenotype was associated with one patient. Both the solid and discohesive organoids showed rapid growth. The discohesive organoids were present in clusters without smooth boundaries.

### ABC enhances the cytotoxic effect of DOX on patient-derived breast cancer organoids

We used the antiretroviral nucleoside analog ABC as a single drug and combined it with DOX to determine its role in promoting DOX-mediated cytotoxicity. Based on our in vitro data and other published work, the breast cancer organoids were exposed to 50 µM ABC and 1 µM DOX, respectively^19,29^. We used the bright-field images to analyze the growth rate (diameter) of the organoids treated with ABC, DOX, and their combination and compared them with untreated breast cancer organoid controls (Fig. 5D). Though the ABC-treated breast cancer organoid looked smaller than the untreated, we did not observe a statistical significance associated with this arm of treatment. DOX showed a substantial decrease in the diameter of breast cancer organoids, suggesting that our model is functional. Moreover, the organoids showed a dark core reminiscent of apoptotic cells.

**Fig. 6:**
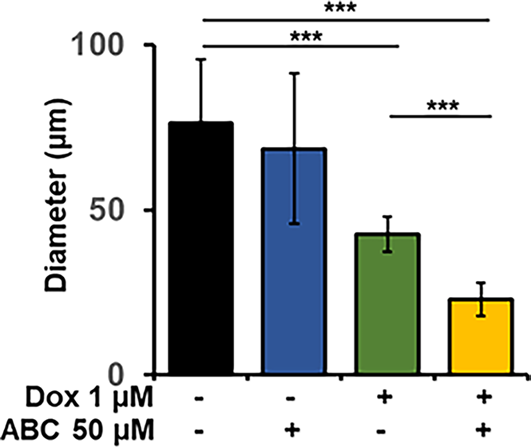
Graph representing the diameter of breast cancer organoids from patients 1 and 2 treated with ABC, DOX, and ABC + DOX and compared with untreated. Data are presented as mean ± standard deviation. Data from patients 1 and 2 are used for plotting the graph. Each experiment was done in triplicate, and 10 spheres per condition were used to measure diameter size. ***P < 0.001.

Interestingly, the combination yielded strong inhibition of breast cancer organoid proliferation (diameter), especially in patients 1 (*n* = 1) and 2 (*n* = 2) with solid, dense organoid phenotypes. Patient 3 (*n* = 3) did not significantly differ in the DOX vs. ABC + DOX combination, suggesting patient genetic diversity. In patient 4 (*n* = 4) with discohesive breast cancer organoid phenotype, the data looked interesting, but it is difficult to ascertain the diameter of each organoid conclusively. Hence, we used data from patients 1 and 2 to plot the diameter of the breast cancer organoids post-treatment to evaluate the sensitivity of ABC, DOX, and ABC + DOX compared to untreated (Fig. 6). Some representative images used for the data analysis are shown in Supplementary Fig. S6 online. The data (Fig. 6) reveal that ABC, in combination with DOX, can be an effective therapeutic option for some patients. Genetic variability can be at the heart of this observation. Because telomerase activity is a promising therapeutic target in breast cancer, and recent reports establish that ABC is functional against telomerase-high medulloblastoma cells, our patient-derived breast cancer organoids can be an interesting strategy for determining the effectiveness of the therapeutic regimen based on ABC and DOX^30^. However, this warrants further investigation.

### Molecular studies reveal CBV-TP interaction with human DNA polymerase β and DNA complex

**Fig. 7:**
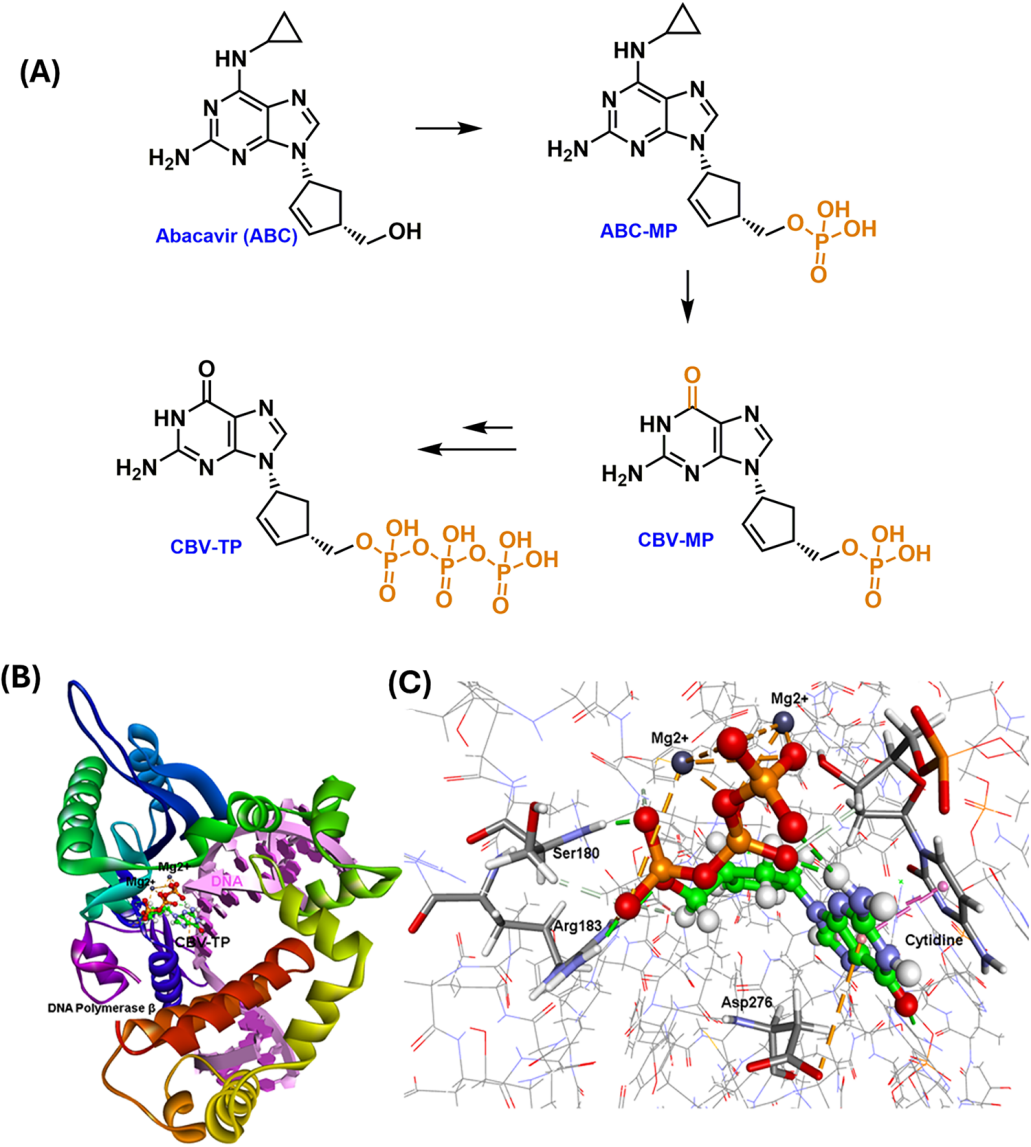
(**A**) ABC is a prodrug that undergoes intracellular bioconversion to form abacavir monophosphate (ABC-MP), carbovir monophosphate (CBV-MP), and the active metabolite carbovir triphosphate (CBV-TP). (**B**) Interactions of CBV-TP (ball and stick cartoon) with human DNA polymerase β (ribbon diagram) and DNA substrate complex; (**C**) Close-up view of CBV-TP interactions with DNA polymerase β and DNA with key interacting residues highlighted.

The results obtained from this study show that ABC pretreatment enhanced the cytotoxicity of DOX to MCF-7 and MDA-MB-231 cells compared to DOX monotherapy. This was an exciting finding from our study. In this regard, there is previous literature that has demonstrated that nucleoside derivatives exhibit anticancer activity by getting incorporated into the DNA synthesis cycle through the DNA polymerase enzymes, including DNA polymerase β^31–33^. For example, the purine nucleoside cladribine is known to exhibit anticancer activity by inhibiting DNA polymerase enzymes^34^. It is pertinent to note that these nucleoside derivatives undergo a series of intracellular bioconversions to form the active triphosphate derivatives. In the case of ABC, it undergoes bioconversion to form the active metabolite carbovir triphosphate (CBV-TP, Fig. 7A)^35^. Therefore, we investigated the interactions of CBV-TP in the DNA polymerase β-DNA complex using the solved X-ray structure^36^.

The top binding mode of CBV-TP in the DNA polymerase β-DNA complex shows that it was oriented in the catalytic site closer to magnesium atoms and the DNA base pairs, as shown in (Fig. 7B). The planar, bicyclic guanosine ring of CBV-TP underwent multiple π-π stacked interactions with the cytosine ring of the DNA nucleotide cytidine (Fig. 7C). Both the imidazole and pyrimidine rings of CBV-TP were in contact with the DNA base (distance < 4.2 Å). Furthermore, the triphosphate ester moiety of CBV-TP underwent polar interactions with amino acids Ser180, and Arg183 of DNA polymerase β (distance < 2.0 Å). Also, it underwent a number of electrostatic interactions with the two catalytic magnesium atoms, as shown in Fig. 7C (distance < 3.5 Å). These molecular docking studies suggest that CBV-TP can interact with the DNA polymerase β-DNA complex and has the potential to get incorporated into the DNA synthesis cycle, causing cell cycle arrest in tumor cells. This is further supported by a previous work that demonstrated the ability of CBV-TP to inhibit DNA polymerases^37^.

## Discussion

Epigenetic modifications, including DNA methylation and histone modifications, play a vital role in cancer development, either by downregulation of tumor suppressor genes or by upregulation of oncogenes^38^. Targeting these mechanisms depicts a promising approach to develop novel anti-cancer drugs^39^. Several studies have emphasized the role of combining epigenetic therapies with conventional treatments, such as chemotherapy and radiation therapy. These combinatorial approaches have proven the ability to enhance therapeutic efficacy, improve sensitivity, and reduce the toxicity associated with conventional therapies^8,40–42^.

The oncogenic significance of KDM5B in breast cancer was confirmed through the analysis of publicly accessible databases. mRNA expression profiles from TCGA datasets revealed significantly higher KDM5B expression levels in breast tumors compared to normal tissues. This finding aligns with our qPCR analysis of 53 breast tumor samples, which confirmed KDM5B overexpression in tumor samples compared to adjacent normal tissues. Our patient cohort exhibited a slightly higher median KDM5B gene expression in luminal subtypes. This is consistent with the established role of KDM5B as a luminal lineage-driving oncogene. Supporting this, Yamamoto et al. demonstrated that high KDM5B activity correlates with poor outcomes in ER + tumors^43^. However, larger patient cohorts are required in our study to establish a significant statistical association with other clinical parameters.

Despite numerous KDM inhibitors being developed in various laboratories, no drugs specifically targeting KDM5B have been approved by the FDA for the management of breast cancer. In this regard, drug repurposing offers an efficient strategy for identifying new therapeutic applications for existing drugs, with fewer time constraints than the traditional drug discovery and development process^14^. More specifically, anti-viral drugs have been shown to sensitize resistant cancer cells to chemotherapy, thereby increasing the efficacy of other therapeutic approaches. For example, ribavirin has been identified as an EZH2 inhibitor^44^, while acyclovir has shown the ability to inhibit the proliferation and colony-forming properties of breast cancer cells^45^. In this context, the current study explored ABC’s repurposing potential for managing breast cancer.

Doxorubicin, an anthracycline derivative that targets topoisomerase II, is widely utilized for various cancers, including breast cancer, carcinomas, sarcomas, and hematological malignancies^46^. Despite the extensive use of doxorubicin, dose-dependent toxicity, particularly cardiotoxicity, remains a significant concern^47^. In this regard, several approaches have been explored to reduce these adverse effects, including the combination of antioxidants, advanced drug delivery systems, and prodrug development^48^. However, some of these approaches have not successfully translated into effective clinical outcomes^48^. Thus, innovative strategies are necessary to enhance therapeutic outcomes. Towards this, our study highlights, for the first time, that epigenetic targeting of KDM5B oncogene by ABC, when combined with DOX, induces cytotoxic effects within a minimal dose range of DOX and may potentially negate the side effects, including cardiotoxicity.

We conducted a series of in vitro experiments to compare the effects of ABC and DOX, both individually as well as in combination, on MCF-7 and MDA-MB-231 breast cancer cell lines. Our results demonstrated that ABC inhibited cell proliferation and induced cytotoxicity. This is in accordance with a previous study showing that ABC causes a dose-dependent decrease in the proliferation rate of medulloblastoma cells^30^. Furthermore, ABC arrests cells in the S phase, consistent with findings reported by Tada et al.^19^. Compared to control, ABC + DOX treatment induced a more pronounced S phase accumulation in MDA-MB-231 than in MCF-7 cells. This might be due to the intrinsic biological and molecular differences between the two cell lines. MDA-MB-231, as a TNBC cell line, exhibits higher genomic instability and impaired G1/S checkpoint control, leading to S phase arrest upon DNA damage^49^. In contrast, MCF-7 cells with intact p53 and checkpoint pathways arrest at the G2/M phase in response to genomic stress^50^.

Our patient-derived breast cancer organoid data suggest that some patients may not be sensitive to this treatment, possibly due to inherent genetic variability. Besides, recent studies have demonstrated the efficacy of ABC against telomerase-high medulloblastoma cells^30^. Therefore, the patient-derived breast cancer organoids developed in this study may be utilized as a precision medicine approach for deciding the treatment regimen based on ABC for breast cancer patients. However, further investigation is needed to validate our findings.

Nucleoside derivatives like ABC exert anti-cancer effects by integrating into the DNA synthesis cycle through DNA polymerase enzymes, such as DNA polymerase β^31^. In cells, ABC is metabolized to its active form, CBV-TP^35^, which is subsequently incorporated into chromosomal DNA by replicative DNA polymerases. This incorporation results in premature termination of DNA replication, replication fork collapse, and the formation of DNA double-strand breaks (DSBs)^19^. Tada et al. confirmed that therapeutic concentrations of ABC induce DSBs in adult T-cell leukemia (ATL) cells, suggesting that efficient induction of DSBs is the primary mechanism underlying ABC’s cytotoxicity^19^. Additionally, a study by Rossi et al. also supports the DNA damage induced by ABC and suggests that its antiproliferative effect is accompanied by the inhibition of telomerase activity in medulloblastoma cell lines^30^. Our computational analysis further supports these findings by demonstrating that CBV-TP interacts with the DNA polymerase β-DNA complex, thereby emphasizing its role in causing cell death by disrupting DNA synthesis in tumor cells.

We also evaluated the impact of ABC treatment on KDM5B expression at both the mRNA and protein levels. Our results show that ABC exposure leads to downregulation of KDM5B in both MDA-MB-231 and MCF-7 cell lines. This effect was particularly evident in MDA-MB-231 cells, reaching statistical significance and suggesting greater sensitivity of MDA-MB-231 to ABC-mediated targeting of KDM5B.

ABC-mediated targeting of KDM5B is supported by the previous study that explored the repurposing potential of ABC through molecular docking studies, targeting the JmjC domain of KDM5B^16^. KDM5B also functions as a genome stabilizer and plays a crucial role in the DNA damage response^51^. Inhibition of KDM5B disrupted the DNA repair mechanism, as demonstrated by Bayo et al., which showed that the small molecule JIB-04 enhanced the sensitivity of lung cancer cells to radiation by inhibiting KDM5B^52^.

In this context, the current study showed increased sensitivity of breast cancer cells towards DOX treatment following ABC pre-treatment. More specifically, our findings suggest that ABC causes DNA damage by being incorporated into the DNA synthesis cycle, leading to the formation of DSB while simultaneously inhibiting KDM5B, which is a critical factor in DNA damage repair. Inhibition of KDM5B exacerbates the accumulation of DNA damage, sensitizing cells to additional DNA-damaging agents such as DOX. Therefore, combining ABC and DOX might amplify the disruption of DNA repair pathways, resulting in enhanced cytotoxicity. These dual actions create a mechanistic synergy, where the inability to repair damaged DNA leads to increased cell death and may also reduce the DOX dosing regimen, potentially lowering its adverse effects. This provides a strong rationale for the combined use of ABC and DOX in breast cancer therapy.

Our study provides foundational data, emphasizing the need for further investigations in animal models or patient-derived xenografts to fully assess the therapeutic potential and translate these findings into clinical settings. Furthermore, studies involving larger patient cohorts are necessary to establish significant correlations between KDM5B expression and clinical outcomes. Comprehensive proteomic and transcriptomic analysis focusing on the epigenetic regulation of KDM5B and its downstream targets will provide deeper mechanistic insights into how ABC disrupts KDM5B-mediated oncogenesis. Further evaluation using other DNA-damaging compounds, along with validation assays, would strengthen the findings. Collectively, these future studies could pave the way for early-phase clinical trials to evaluate the safety, tolerability, and preliminary efficacy of ABC as an adjuvant to DOX in breast cancer treatment.

## Conclusions

The current study highlights the over-expression of KDM5B in human breast tumors compared to non-neoplastic breast tissues in global databases as well as in our patient cohort. In vitro studies showed that ABC treatment reduced KDM5B expression in breast cancer cells and increased their sensitivity towards DOX treatment. ABC induced late apoptosis and S-phase arrest, while the ABC + DOX combination led to S/G2 phase arrest, late apoptosis, and cell death. Drug treatment studies in the patient-derived breast tumoroids supported the 2-D cell line-based findings. Additionally, molecular docking studies indicated that CBV-TP could interact with the DNA polymerase β-DNA complex, suggesting its potential mechanism to be incorporated into the DNA synthesis cycle, leading to cell cycle arrest in tumor cells. Our findings highlight the repurposing potential of ABC, a well-established antiviral agent, as a novel therapeutic approach targeting the KDM5B oncogene in breast cancer. This strategy may enhance the efficacy of DOX and could potentially help in reducing its dose, side effects, and drug resistance, thereby offering a promising approach for personalized breast cancer treatment.

## Methods

### KDM5B gene expression across various cancer types

The expression profile of KDM5B across all cancer types was obtained from Gene Expression Profiling Interactive Analysis (GEPIA)^21,22^. The University of Alabama at Birmingham Cancer Data Analysis Portal (UALCAN) was used to retrieve KDM5B expression data based on various clinical and histopathological factors^23–25^. The TNMplot database was used to evaluate KDM5B expression in breast tumors, normal and metastatic tissues^26,27^. Additionally, overall survival (OS) in breast cancer patients was assessed in relation to KDM5B mRNA expression using GEPIA^21,22^.

### Patient enrolment and sample collection

Paired breast tumors and adjacent normal tissues were collected from newly diagnosed breast cancer patients aged 18 to 80 years who underwent breast conservative surgery or modified radical mastectomy at Kasturba Medical College, Manipal, India. Ethical approval for the study was granted by the Kasturba Medical College and Kasturba Hospital Institutional Ethics Committee (IEC: 504/2019), and written informed consent was obtained from all participants. The research was performed in accordance with the Declaration of Helsinki. Clinical samples were collected in RNAprotect reagent (Qiagen, Hilden, Germany) and stored at -80 °C until further use. A certified pathologist characterized all tumor samples, and clinical parameters were retrieved from medical records.

### RNA isolation and cDNA synthesis

Total RNA was extracted using the RNeasy Protect Mini Kit (Qiagen, Germany). According to the manufacturer’s protocol, an on-column DNase digestion step was performed with the RNAse-Free DNase Set (Qiagen, Germany). The concentration and purity of the RNA were assessed using a NanoDrop^®^ ND-1000 spectrophotometer (Thermo Scientific, USA). A total of 1 µg of RNA from each sample was reverse transcribed into cDNA using the iScript™ cDNA Synthesis Kit (BioRad, Hercules, CA, USA).

### Real-time quantitative polymerase chain reaction (qPCR)

After assessing RNA quality and identifying outliers, qPCR was performed on 53 breast tumor samples and 14 unpaired breast normal tissues. The TaqMan gene expression assay kit for KDM5B (Assay ID: Hs00981910_m1, Thermo Fisher Scientific, OH, USA) was used, with beta-actin (Assay ID: Hs99999903_m1, Thermo Fisher Scientific, OH, USA) serving as the internal control. The reactions were performed using the TaqMan^®^ Fast Advanced Master Mix (Thermo Fisher Scientific, OH, USA) on a QuantStudio 5 Real-Time PCR (Applied Biosystems, USA) under standard cycling conditions. Ct values were normalized to beta-actin, and relative expression in tumor samples was determined using normal breast tissue samples as the control. The fold change in gene expression was calculated using the 2^− ddCt^ method^53^.

### Cell culture

Human breast cancer cell lines, MDA-MB-231 (ATCC, Manassas, Virginia) and MCF-7 (ECACC, UK), were cultured at 37 °C, 5% CO_2_ in minimum essential media (MEM, Gibco™), supplemented with 10% heat-inactivated fetal bovine serum (FBS, Gibco™), 1% non-essential amino acids (Gibco™) and 1% penicillin/streptomycin (Pen Strep, Gibco™). For subculturing, the media was aspirated, and the cells were washed with PBS (pH 7.4). Cells were passaged using 0.25% Trypsin-EDTA (Thermo Fisher Scientific, OH, USA) for 5 min, and the trypsinization was inhibited by adding complete media. The cells were then centrifuged and resuspended in fresh media for subsequent subculture.

### Drug treatment

ABC was received from Hetero Labs Limited and Mylan Laboratories Limited, while CBV (> 95% pure) was sourced from Biosynth International, Kentucky, USA. Pharmaceutical-grade DOX was procured from Fresenius Kabi India Pvt. Ltd., India. All compounds were dissolved in sterile water.

MDA-MB-231 and MCF-7 cells were seeded into 96 well plates at a density of 5 × 10^3^ cells/well. The next day, the cells were treated with ABC and CBV across a concentration range of 0 to 500 µM for 72 h with daily replenishment to determine the half-maximal inhibitory concentration (IC_50_) in monotherapy. To assess the influence of ABC on KDM5B mRNA and protein expression, cells were treated with ABC at its IC_25_ concentration for 24 h. Based on this, for combination therapy, cells were pre-treated with low-dose ABC (IC_25_) for 24 h, followed by DOX treatment at concentrations ranging from 0 to 160 nM every 24 h for three days. For further 2-D in vitro assays, four treatment groups were included: (1) Control (no treatment), (2) ABC-only treatment (IC_25_ dose for three days), (3) DOX-only treatment (IC_25_ dose for 3 days), and (4) ABC + DOX (ABC pre-treatment at IC_25_ dose for 24 h, followed by DOX treatment at IC_25_ dose for three days).

### MTT assay

Cell viability was assessed using MTT (3-(4,5-dimethylthiazol-2-yl)-2,5 diphenyl tetrazolium bromide) assay. Briefly, MDA-MB-231 and MCF-7 cells (5000 cells/well) were seeded in triplicate in 96-well plates and treated with increasing concentrations of drugs (0 to 500 µM) for 72 h with daily replenishment. Following treatment, MTT dye (0.5 mg/ml) was added, and cells were incubated for 4 h. Formazan crystals were dissolved in DMSO, and absorbance was measured at 570 nm using an ELISA microplate reader. The IC_50_ values were determined using Prism software (GraphPad Software, La Jolla, CA).

To evaluate the ability of ABC to enhance DOX sensitivity, MDA-MB-231 and MCF-7 cells were pre-treated with ABC at its IC_25_ concentration for 24 h. Subsequently, cells were exposed to DOX concentrations ranging from 0 to 160 nM for 72 h, with daily replenishment. The MTT assay was performed, and the IC_50_ values of DOX in ABC-sensitized cells were compared to those in cells treated with DOX alone.

### Immunofluorescence

For immunofluorescence analysis, cells were seeded on tissue culture polystyrene dishes and cultured for 24 h until 70% confluence. Cells were treated as per the respective drug treatment conditions mentioned above. Following treatment, cells were fixed in 4% paraformaldehyde and were blocked and permeabilized with 0.1% Triton X-100 and 2.5% goat serum for 1 h. After washing with PBS, cells were incubated overnight at 4 °C in a humidified chamber with primary antibodies: KDM5B antibody (Cat. No: A301813A, JARID1B polyclonal antibody, Thermo Fisher Scientific) or cleaved caspase-3 antibody (Cat. No: 9661, Cell Signalling Technology) as required. The cells were then washed with PBS and incubated with fluorescent-labelled goat anti-rabbit IgG secondary antibody (Cat. No: A-11008, Thermo Fisher Scientific). Nuclei were counterstained using DAPI (Cat. No: D1306, Thermo Fisher Scientific), and the samples were mounted using an antifade mounting reagent, followed by imaging using a fluorescence microscope (Nikon Ti-S Eclipse). The images were processed and quantified using the Image J software, and the quantified data were further analyzed using GraphPad Prism software version 8.0.2.

### Soft agar colony forming assay

A 24-well plate was prepared with a base layer of 0.6% agar in 2X complete media and allowed to solidify. The top agar layer (0.3%), containing 2000 cells from the respective treatment groups, was seeded onto the solidified base layer and allowed to set. The plates were incubated at 37 °C with 5% CO_2_ to promote colony formation. To maintain cell growth, 100 µl of media was added to each well every three days. The assay was completed on day 28, and the colonies were stained with 0.1% crystal violet solution. Stained colonies were then observed and counted^54^.

### Apoptosis assay

The apoptosis study was performed using FITC-Annexin V Apoptosis Detection Kit I (BD Pharmingen™, USA) following the manufacturer’s instructions^55^. Briefly, pretreated cells at a concentration of 1 × 10^6^ cells/ml were washed with cold PBS and re-suspended in the 1X binding buffer provided in the kit. A 100 µl aliquot of this cell suspension (1 × 10^5^ cells) was stained with 5 µl FITC Annexin V and 5 µl propidium iodide (PI) and then incubated for 15 min at room temperature in the dark. 400 µl of 1X binding buffer was added to the stained cells and analyzed for apoptosis using BD FACSAria™ Fusion Flow cytometer (Becton, Dickinson and Company, USA). The results were analyzed using FlowJo™ v10.8 Software (BD Life Sciences, USA).

### Cell cycle analysis

Pretreated cells were harvested and fixed in ice-cold 70% ethanol for 30 min. After fixation, the cells were rinsed with PBS and incubated with RNase A (100 µg/ml, Thermo Fisher Scientific, USA) for 30 min at room temperature. Subsequently, the cells were then stained with PI at a concentration of 50 µg/ml and incubated for an additional 30 min at room temperature in the dark. Flow cytometric analysis was performed immediately using the BD FACSAria™ Fusion Flow cytometer (Becton, Dickinson and Company, USA), with a minimum of 10,000 cells collected per sample. Data were analyzed using FlowJo™ v10.8 Software (BD Life Sciences, USA). Appropriate controls, including unstained and single-stained cells, were used to set compensation and gating parameters^56^.

### 3-D breast cancer organoid culture

Breast cancer tissues were cut into 1-3 mm^3^ pieces and processed to isolate viable cells. The tissue was minced and washed with 10 ml of collection medium (Advanced DMEM/F12 with 1X Glutamax, 10 mM HEPES, and antibiotics). The tissue was digested in ~ 5-10 ml breast cancer organoid medium containing 2 mg/ml collagenase (Sigma-Aldrich, St. Louis, MO) and mixed on an orbital shaker at 37 °C for 2 h. The processed tissue suspension was subjected to successive shearing using sterilized glass Pasteur pipettes. After each shearing step, the suspension was strained through a 100 μm filter to retain larger tissue fragments. The fragments were further subjected to additional shearing with a 10 ml collection medium. To the strained suspension, 2% FBS was added, followed by centrifugation at 400 rcf. The resulting pellet was resuspended in a 10 ml collection medium and centrifuged at 400 rcf. If a visible red pellet was observed, erythrocytes were lysed using 2 ml of red blood cell lysis buffer (Cat. No: 11814389001, Roche Diagnostics, Germany) for 5 min at room temperature, followed by the addition of 10 ml of collection media and centrifugation at 400 rcf.

The final pellet was resuspended in cold growth factor reduced Matrigel (Corning, USA) (1:1 of media and matrigel), and 100 µl of the suspension was plated in transwell chambers. 500 ml of breast cancer organoid medium was added to each well, and plates were incubated at 37 °C, 5% CO_2_. The breast cancer organoid medium contained R-Spondin 3 (Sigma-Aldrich, St. Louis, MO), Neuregulin 1, FGF 7, FGF10, and EGF (Thermo Fisher-PeproTech), along with Rock Inhibitor, HEPES, and N-Acetylcysteine (Sigma-Aldrich, St. Louis, MO)^28^. The medium was changed every 2 days, and organoids were passaged every 2-4 weeks. For passaging, dispase (Sigma-Aldrich, St. Louis, MO) was added to the transwells and incubated for 2-3 h. Organoids were dissociated by incubating in 2 ml of TrypLE Express (Gibco, Thermo Fisher) at 37 °C for 3-5 min, followed by mechanical shearing with sterilized glass Pasteur pipettes. After adding 10 ml of collection medium, the suspension was centrifuged at 400 rcf, and the resulting pellet was harvested to prepare a single-cell suspension. The cells were seeded at high density and reseeded at reduced density after the first passage. Organoid culture was performed by following Hans Clevers group’s recent protocol^28,57^. Once the diameter of breast cancer organoids reached around 20-30 μm, they were subjected to doses of ABC (50 µM at 24, 48, 72, and 96 h) and DOX (1 µM at 48, 72, and 96 h), and a combination of ABC + DOX (24, 48, 72, and 96 h). The overall shape and dimensions of the breast cancer organoids were analyzed using a brightfield microscope. Three sets of experiments were conducted per patient (*n* = 3 per patient), and ten representative organoids per condition were measured^58,59^. Organoid sizes were determined using Image J (OpenJDK 13.0.6.)^59,60^.

### In silico molecular studies

The binding interactions of CBV-TP in the human DNA polymerase β enzyme in complex with DNA were studied using the computational software Discovery Studio (DS) Structure-Based-Design (BIOVIA Inc, Dassault Systemes, USA). The CBV-TP was built in 3D using the Small Molecules module in the software and was energy minimized over 2000 steps using the Smart Minimizer, distance-dependent dielectrics, and CHARMm force field. The X-ray structure of human DNA polymerase β enzyme in complex with DNA and the nucleotide derivative 2’-deoxyuridine-5’-[(a, b)-imido] triphosphate (dUMPNPP) was obtained from PDB (PDB ID: 2FMS)^36^. The protein-DNA-nucleotide complex was prepared first, by removing water molecules. The two magnesium atoms (Mg^2+^) in the catalytic site were retained. The bound nucleotide dUMPNPP was selected, and a binding site sphere of 8 Å was created around the nucleotide dUMPNPP. In the next step, the nucleotide was deleted. Subsequently, the Macromolecules module in the software was used to prepare the protein-DNA complex using the CHARMm force field. Molecular docking was carried out using the LibDock algorithm in DS with the following parameters: 100 hotspots, docking tolerance of 0.25 Å, implicit solvent function, and distance-dependent dielectric constant. The docking poses were energy minimized over 1000 steps using the Smart Minimizer. The CHARMm force field was used during the docking simulation. The docking poses were ranked based on the LibDock score and were analyzed by investigating the polar and nonpolar interactions of CBV-TP with human DNA polymerase β enzyme and DNA.

### Statistical analysis

The normality of distribution was tested using the Shapiro-Wilk test. Continuous variables were reported as either the median with an interquartile range or the mean with standard deviation, depending on the normality of the data. Differences in normally distributed data were analyzed using Student’s t-test or one-way analysis of variance (ANOVA). The Wilcoxon Mann-Whitney test was used to compare the differences in gene expression levels between tumor and normal samples. Overall survival rates were estimated using the Kaplan–Meier method, with the median KDM5B expression level taken as the cutoff for high and low expression groups. All statistical analyses were performed using GraphPad Prism version 8.0.2 (GraphPad Software Inc., San Diego, CA, USA), with *P* < 0.05 considered statistically significant.

## Supplementary Information

Below is the link to the electronic supplementary material.

Supplementary Material 1

## Data Availability

All data supporting the findings of this study are available within the paper and its Supplementary Information.
