# Supplementary material for: Abacavir enhances the efficacy of doxorubicin via inhibition of histone demethylase KDM5B in breast cancer
Source: Sci Rep. 2025 Aug 5;15:28531. doi: 10.1038/s41598-025-13845-z (PMC12325968; doi:10.1038/s41598-025-13845-z)
Supplement: Supplementary file 1 — Supplementary Material 1 [file 41598_2025_13845_MOESM1_ESM.pdf]

Supplementary File

Abacavir enhances the efficacy of Doxorubicin *via* inhibition of histone demethylase KDM5B in breast cancer

Anmi Jose[1], Pallavi Kulkarni[2], Naveena Kumar AN[3], Nawaz Usman[3], Gabriel Sunil Rodrigues[4], Gautham G Shenoy[5], Rama Rao Damerla[6], Murali Munisamy[7], Bharti Bisht[8], Sooryanarayana Varambally[9], Manash K Paul[10], Neha Arya[7], Praveen PN Rao[11], Mahadev Rao*[1]

1. Department of Pharmacy Practice, Manipal College of Pharmaceutical Sciences, Manipal Academy of Higher Education, Manipal 576104, Karnataka, India
2. Department of Biochemistry, All India Institute of Medical Sciences, Bhopal, 462020, Bhopal, Madhya Pradesh, India
3. Department of Surgical Oncology, Manipal Comprehensive Cancer Care Centre, Kasturba Medical College, Manipal Academy of Higher Education, Manipal 576104, Karnataka, India
4. Department of General Surgery, Kasturba Medical College, Manipal Academy of Higher Education, Manipal 576104, Karnataka, India
5. Department of Pharmaceutical Chemistry, Manipal College of Pharmaceutical Sciences, Manipal Academy of Higher Education, Manipal 576104, Karnataka, India
6. Department of Medical Genetics, Kasturba Medical College, Manipal Academy of Higher Education, Manipal 576104, Karnataka, India
7. Department of Translational Medicine, All India Institute of Medical Sciences, Bhopal, 462020, Bhopal, Madhya Pradesh, India
8. Department of Microbiology, Kasturba Medical College, Manipal Academy of Higher Education, Manipal 576104, Karnataka, India
9. Department of Pathology, University of Alabama at Birmingham, Birmingham, AL, USA

10. Department of Radiation Biology and Toxicology, Manipal School of Life Sciences, Manipal Academy of Higher Education, Manipal 576104, Karnataka, India
11. School of Pharmacy, Health Sciences Campus, 200 University Avenue West, University of Waterloo, Waterloo, ON N2L 3G1, Canada

***Corresponding author**: Dr. Mahadev Rao, Professor, Department of Pharmacy Practice, Coordinator, Centre for Translational Research, Manipal College of Pharmaceutical Sciences, Manipal Academy of Higher Education, Manipal 576104, Karnataka, India.

Email: mahadev.rao@manipal.edu

1. Supplementary Figure 1

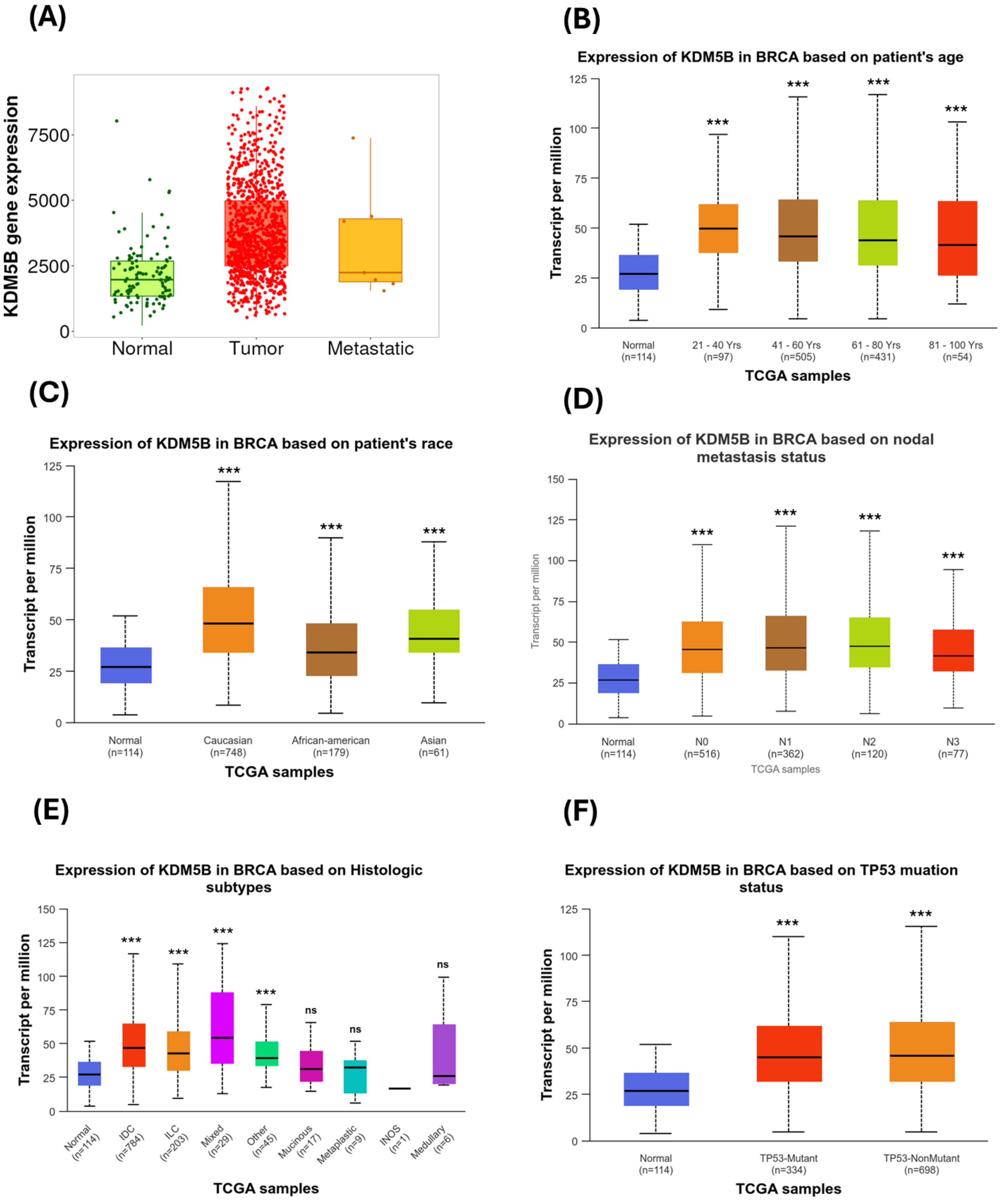

Fig S1: KDM5B expression and its correlation with other clinicopathological parameters; **(A)** KDM5B gene expression analysis using RNA-Seq data from TNMplot database in breast cancer

tumor (n=1097), normal (n=113), and metastatic tissue (n=7) (*P* value < 0.001, Kruskal-Wallis test); **(B-F)** UALCAN analysis of expression profiles of KDM5B in breast cancer across different age groups, patient's race, nodal metastasis, histological subtypes, and TP53 mutation status. *$P < 0.05$; **$P < 0.01$; ***$P < 0.001$; ns: not significant.

2. Supplementary Figure 2

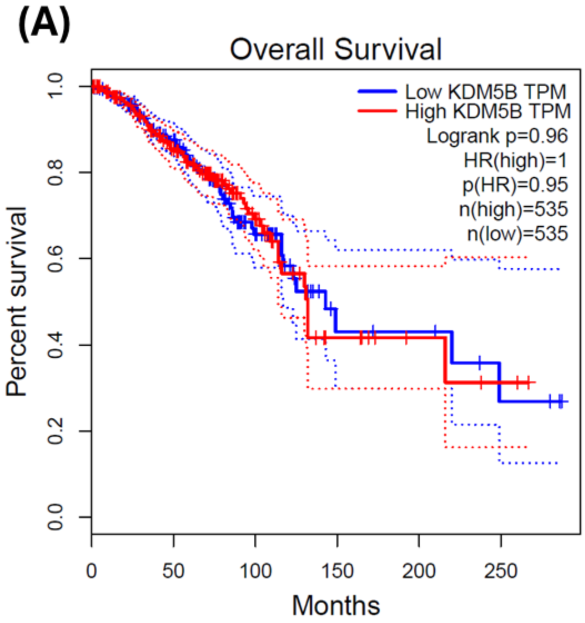

Fig S2: Overall survival in breast cancer patients in relation to KDM5B mRNA expression; **(A)** OS in breast cancer patients in relation to mRNA expression was assessed in silico through GEPIA. Kaplan–Meier plots and log-rank test *P*-values were generated computationally. Patient groups were stratified based on median KDM5B gene expression values extracted from TCGA-BRCa datasets.

3. Supplementary Figure 3

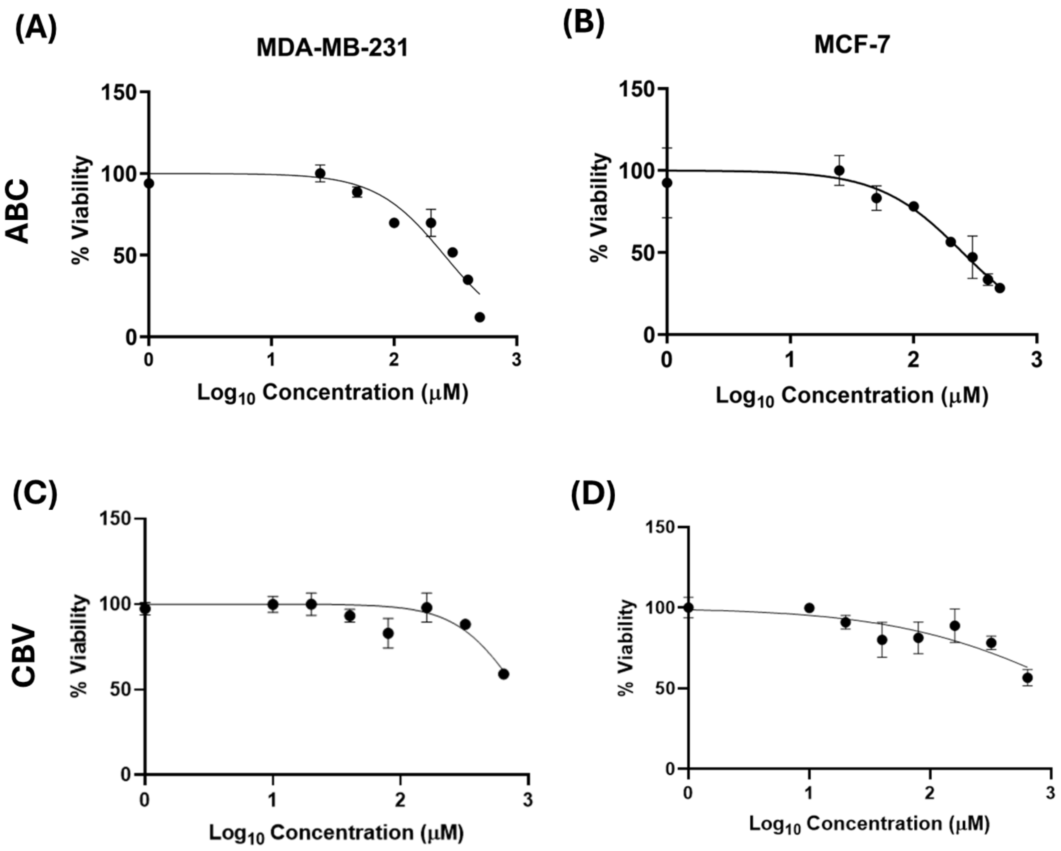

	IC50 values (μM)	
	ABC	CBV
MDA-MB-231	289.15 ± 32.25	>500
MCF-7	281.26 ± 51.59	>500

Fig S3: Cytotoxicity of ABC and CBV in MDA-MB-231 and MCF-7 cell lines; Representative images for the ABC and CBV cytotoxicity study by MTT assay. IC_{50} values represent three independent experiments, expressed as mean ± SD.

4. Supplementary Figure 4

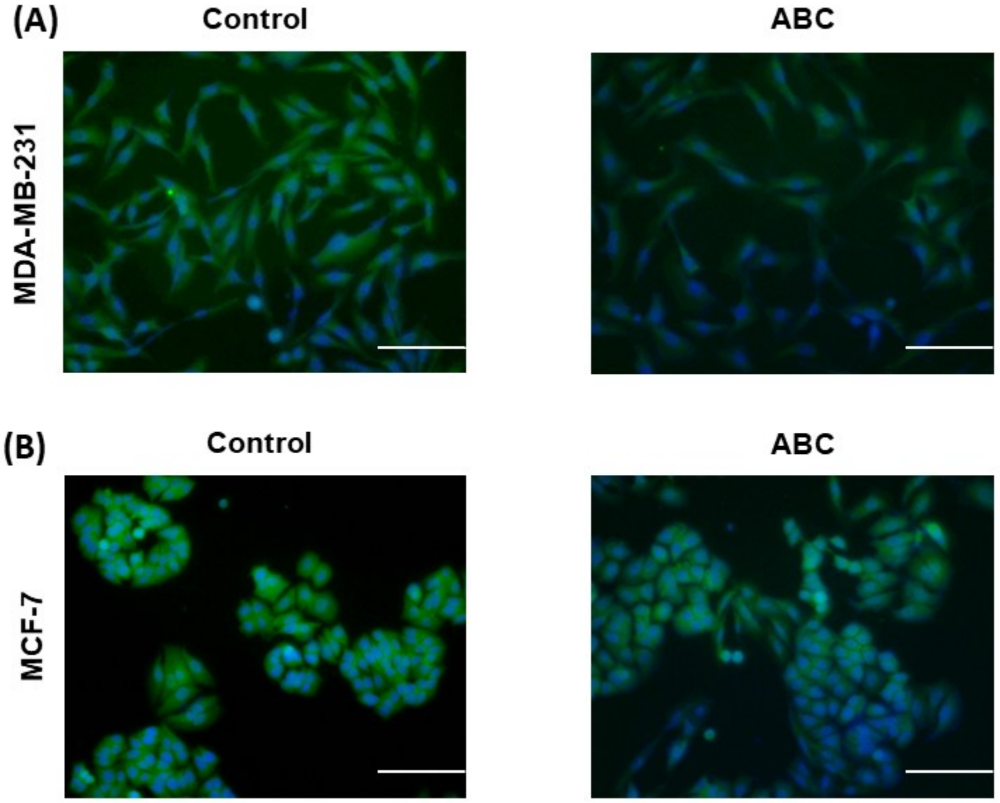

Fig S4: Immunofluorescence staining of KDM5B protein in control and ABC-treated cells. **(A-B)** Representative images of control and ABC-treated MDA-MB-231 and MCF-7 cells. Fluorescence intensity was normalised to DAPI. Data are presented as mean ± standard deviation from three independent experiments. Scale bar: 500 μm.

5. Supplementary Figure 5

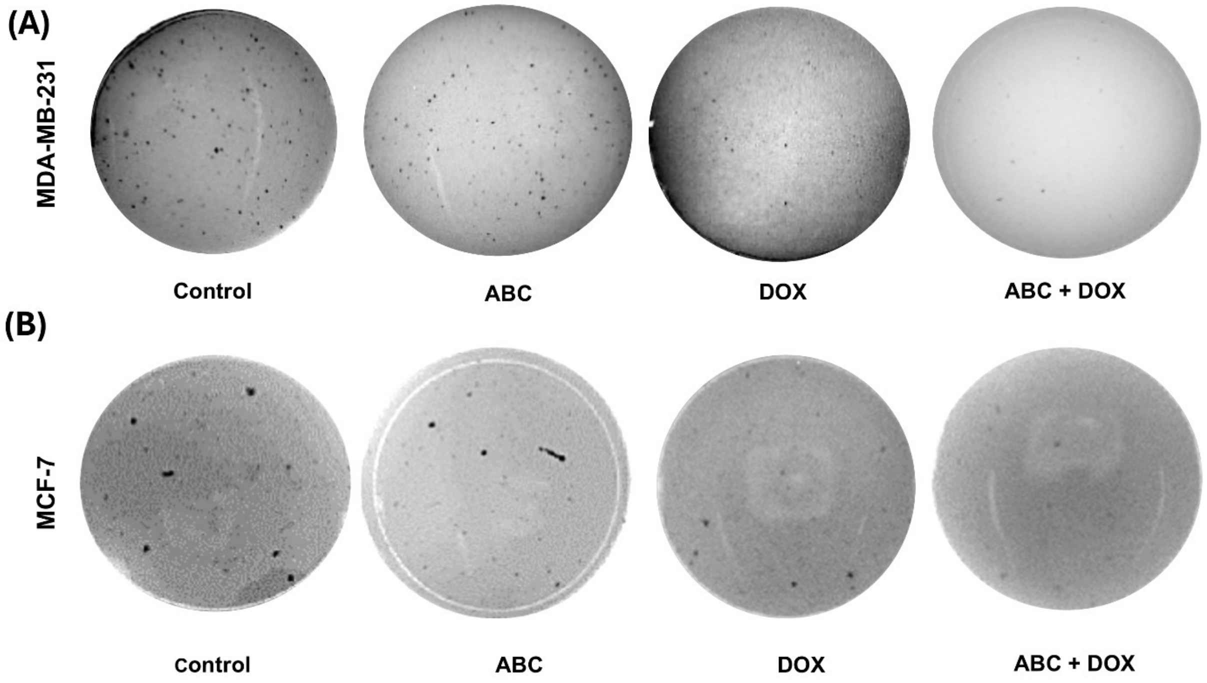

Fig S5: Soft agar colony forming assay. Images of soft agar wells colony formation for treatment groups **(A)** MDA-MB-231 and **(B)** MCF-7**.**

6. Supplementary Figure 6

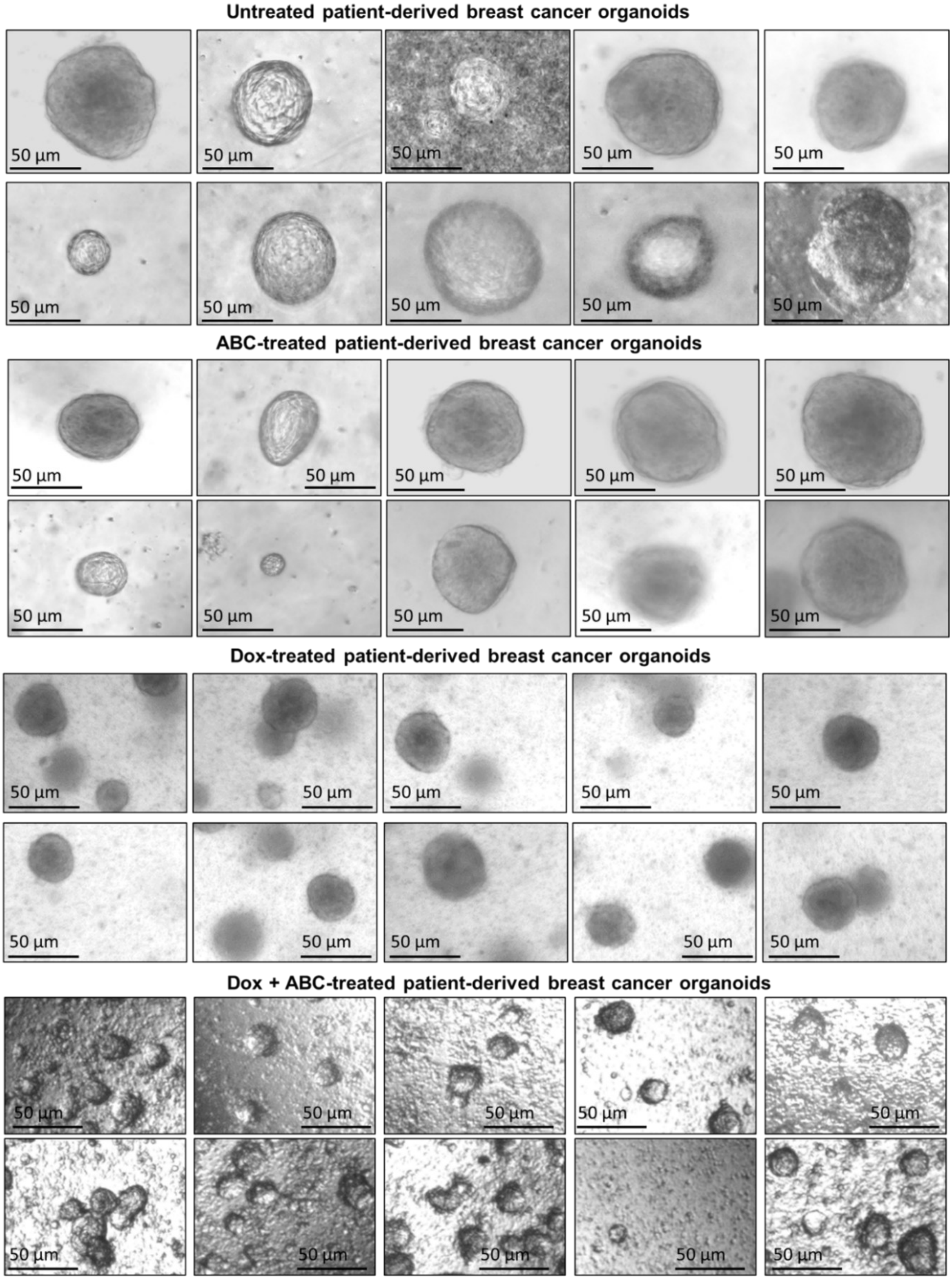

Fig S6: Patient-derived breast cancer organoids. Representative bright-field images of patient-derived breast cancer organoids used for data analysis are shown.
